# Extensive clinical target volume in postoperative chemoradiotherapy for esophageal squamous cell carcinoma: a phase II clinical trial (ESO-Shanghai 9)

**DOI:** 10.1186/s13014-023-02211-w

**Published:** 2023-02-07

**Authors:** Dashan Ai, Yun Chen, Qi Liu, Jiaying Deng, Xiaofei Zhang, Junhua Zhang, Li Chu, Jingyi Shen, Longfei Ma, Yawei Zhang, Haiquan Chen, Longsheng Miao, Kuaile Zhao, Jiaqing Xiang

**Affiliations:** 1 Department of Radiation Oncology, Fudan University Shanghai Cancer Center, Shanghai, 200032 China; 2 Department of Oncology, Shanghai Medical College, Fudan University, Shanghai, 200032 China; 3 Department of Thoracic Surgery, Fudan University Shanghai Cancer Center, Shanghai, 200032 China

**Keywords:** Esophageal cancer, Postoperative treatment, Radiotherapy, Chemotherapy

## Abstract

**Background:**

To compare the efficacy and safety of postoperative extensive target volume irradiation with elevated radiation dose and concurrent chemotherapy with radiotherapy only for the postoperative treatment of esophageal squamous cell carcinoma.

**Methods:**

This trial was a single-arm phase II trial. Patients who underwent a radical transthoracic resection with negative margins within 3 months and histologically confirmed esophageal squamous cell carcinoma (pT3-4N0M0 or pTxN + M0, AJCC 7th) were eligible for this study. Postoperative radiotherapy was performed at a total dose of 45 Gy in 25 fractions with clinical target volumes of the tumor bed, anastomosis, bilateral supraclavicular, mediastinal, left gastric and celiac trunk lymph node areas. Five cycles of weekly TC (paclitaxel 50 mg/m^2^, d1, carboplatin AUC = 2, d1) were given as concurrent chemotherapy. The primary endpoint was the 2-year local control rate, and the secondary endpoints were overall survival, disease free survival, local-regional recurrence free survival, distant metastasis free survival and adverse events. All endpoints were compared with those in ESO-Shanghai 8 study with postoperative radiotherapy alone (40 Gy/20Fx).

**Results:**

A total of 70 patients were enrolled from 2016 to 2018. The 2-year local control rate was 87.9% (95% CI: 83.3–92.3) in this study, which achieved the hypothesized 2-year local control rate of at least 83%. Overall survival, disease free survival, local-regional recurrence free survival and distant metastasis free survival in this study were also longer than those in previous ESO-Shanghai 8 study while most toxicities were increased and two patients in this study died of radiation pneumonitis.

**Conclusions:**

Postoperative extensive target volume irradiation with elevated radiation dose and concurrent chemotherapy was effective. Treatment related toxicity was increased due to higher treatment intensity.

*Trial registration* clinicaltrials.gov: NCT02916511.

**Supplementary Information:**

The online version contains supplementary material available at 10.1186/s13014-023-02211-w.

## Background

Worldwide, esophageal cancer remains one of the most common cancers and the fourth cause of cancer-related death [1]. Preoperative chemoradiotherapy followed by surgery is the standard treatment for operable esophageal cancer, but surgery without neoadjuvant treatment is still common in China. However, over half of patients were found recurrence or metastasis after radical esophagectomy alone [2, 3]. Therefore, postoperative therapy was expected to reduce the recurrence rate and prolong the survival time for patients with surgery alone.

In recent years, series of clinical trials and large sample size retrospective studies demonstrated that postoperative radiotherapy could play a positive role in esophageal cancer, especially for patients with advanced diseases. Xiao et al. reported that among stage III (T4N0-1M0 or T3N1M0, AJCC 6th) patients, 5-year survival rate of postoperative radiotherapy group was 35.1%, which was much higher than 13.1% in the surgery only group. Additionally, postoperative radiotherapy could reduce the incidence of mediastinal and supraclavicular lymph node recurrence [4]. Similarly, in Chen’s study, postoperative radiotherapy was associated with better survival for patients with node-positive esophageal cancer treated with radical esophagectomy with three-field lymphadenectomy [5]. Other large sample size studies based on the Surveillance Epidemiology and End Results (SEER) database and National Cancer Data Base (NCDB) from western countries also proved that in regional esophageal cancer, there was significant improvement in overall survival [6–8].

In the clinical practice of postoperative radiotherapy, the target volumes were various. Xiao et al. set the target volume from bilateral supraclavicular areas to the entire mediastinum and site of anastomosis. As a result, postoperative radiotherapy significantly reduced the recurrence rate of the supraclavicular and mediastinal lymph nodes, while the recurrence rate of abdominal lymph nodes was still high, which was largely attributed to uninvolved the abdominal lymph node area into the irradiation field [4, 9]. Therefore, we performed a clinical trial (ESO-Shanghai 8) to explore the feasibility of extensive target volume in postoperative radiotherapy in esophageal cancer [10]. In this study, tumor bed, anastomosis site, bilateral supraclavicular region, all mediastinal lymph node sites, and left gastric and celiac trunk lymph node area were included in the target volume to cover all high-risk recurrence areas, and the dose was 40 Gy, which was relatively low for safety reasons. The extensive target volume irradiation was feasible in dose distribution and the toxicity was tolerable, but the in-field recurrence rate remained high. We believe the inconsistent result with other studies may be caused by the relatively low dose. Increasing irradiation dose may reduce in-field recurrence rate, thereby improving disease-free survival.

In the research of postoperative treatment, adjuvant chemotherapy was also a key point. In JCOG 9204 study, postoperative chemotherapy could prolong the disease-free survival in N+ patients [11]. Besides, in Chen’s retrospective study, postoperative chemoradiation was significantly more effective than radiotherapy alone at increasing the overall survival and decreasing the rates of distant metastasis and overall recurrence for node-positive patients [12]. In our previous study (ESO-Shanghai 8), the rate of distant metastasis was also high, which greatly affected disease-free survival. Therefore, the addition of postoperative adjuvant chemotherapy into radiotherapy may contribute to prolonging the disease-free survival.

Based on the reasons above, we designed a single-arm, phase II clinical trial, in which we elevated the total dose to 45 Gy and added concurrent chemotherapy, aiming to improve the local control rate and prolong the survival.

## Material and methods

This study was a single-arm phase II clinical trial (NCT02916511), which was initiated in 2016 and finished in 2018 at Fudan University Shanghai Cancer Center. The protocol was approved by the Ethics Committee of Fudan University Shanghai Cancer Center.

### Eligibility

Patients who underwent a radical transthoracic resection with negative margins within 3 months and histologically confirmed esophageal squamous cell carcinoma (pT3-4NxM0 or pTxN + M0 according to AJCC 7th edition) were eligible for this study. No preoperative radiation therapy or chemotherapy was allowed. Locoregional recurrent disease or distant metastases before enrollment should be excluded. Other eligible criteria were as follows, age ≤ 75 years, Karnofsky Performance Status Score ≥ 80, neutrophil count ≥ 1.5 × 10^9^/L, leukocyte count ≥ 3 × 10^9^/L, platelet count ≥ 100 × 10^9^/L, serum creatinine level < 1.5 upper limit of normal (ULN) and ALT or AST level < 2.5 ULN. All participants provided written informed consent.

### Interventions

Patients who met the eligible criteria received concurrent chemoradiotherapy. Radiotherapy began on day 1, concurrent with the beginning of cycle 1 of chemotherapy. Radiotherapy was delivered with photons (≥ 6 MV) from a linear accelerator to a total dose of 45 Gy in 25 fractions. Patients were treated 5 days per week at 1.8 Gy/day. Intensity modulated radiotherapy was required. The definition of clinical target volume (CTV) included tumor bed, anastomosis site, bilateral supraclavicular region, all mediastinal lymph node site, left gastric and celiac trunk lymph node site. The superior, inferior, anterior, posterior and lateral borders of planning target volume (PTV) were 1 cm beyond CTV. The field next to the spinal cord could be slightly adjusted in order to reduce the exposure of spinal cord. (Additional file 1: Fig. S1).

When formulating the treatment plan, normal organ dose restrictions should be taken into consideration (Additional file 2: Table S1). As for target volumes, tissue inhomogeneity correction was adopted and it was required that more than 99% PTV received 95% prescription dose and more than 95% PTV received 99% prescription dose.

All patients were planned to be treated with 5 cycles of weekly chemotherapy concurrent with radiotherapy. The chemotherapy regimen consisted of paclitaxel (50 mg/m^2^) on day 1 and carboplatin (AUC = 2) on day 1.

Patients were followed-up at least weekly in the course of postoperative chemoradiotherapy to monitor the adverse events. If grade 3 or higher hematological and non-hematological toxicities was observed, radiotherapy had to be suspended until toxicity no more than grade 2. If neutrophil count < 1.5 × 10^9^/L or platelet count< 100 × 10^9^/L or grade 2 or higher non-haematological toxicity was observed on day 1 of each cycle, chemotherapy had to be suspended until toxicity no more than grade 1. It was allowed to suspend at most 2 weeks, otherwise radiotherapy or chemotherapy should be terminated.

After the completion of all treatment, patients were evaluated for evidence of local recurrence and distant metastasis every 3 months within the first 2 years, every 6 months for the next 3 years and once a year thereafter. Chest CT scan with contrast, neck and abdomen ultrasound, esophagography (barium swallow) should be processed as routine and esophagoscopy when necessary. Investigators reviewed all the results of the examinations above and made assessments.

### Outcomes

The primary endpoint was 2-year local control rate in all enrolled patients. Local control was defined as no recurrence in the esophageal anastomosis or lymph nodes in the radiation field. The secondary endpoints included overall survival (OS), disease free survival (DFS), local-regional recurrence free survival (LRFS), distant metastasis free survival (DMFS) and adverse events. Overall survival was defined as time from the date of surgery until death. DFS was defined as the time from the date of surgery to the date of recurrence, metastasis or death, whichever occurred first. LRFS was defined as the time from the date of surgery to the date of local-regional recurrence or death, whichever occurred first. DMFS was defined as the time from the date of surgery to the date of distant metastasis or death, whichever occurred first. Toxicity was graded according to the National Cancer Institute Common Terminology Criteria for Adverse Events (NCI-CTCAE 4.0).

### Statistics

In our previous study (ESO-Shanghai 8), the 2-year local control rate was 69.4% in the preliminary results when design of this study in September, 2016. Our hypothesis was the 2-year local control rate was over 83% with the elevated radiotherapy dose and the addition of concurrent chemotherapy. A total of 70 patients were needed to test this hypothesis with a one-sided type I error of 5%, a power of 80%, and a dropout rate of 10%.

Survival was estimated with Kaplan–Meier method. Log-rank test was used to compare the survival and Pearson’s χ^2^ was used to compared the toxicities and treatment completion between this study and our previous study (ESO-Shanghai 8).

A *P* value of less than 0.05 was considered significant. All data analyses were performed using SPSS 22.0.

## Results

### Clinical characteristics

Seventy patients were enrolled in this phase II study from 2016 to 2018, and clinical characteristics were listed in Table 1. Most patients in this study were male (88.6%) and median age of all patients was 60 years. Among all surgical procedures, two-field lymphadenectomy was the most widely used (70%). After restage all the patients in this study, the majority of patients were stage IIIb (60.0%).

**Table 1:** Clinical characteristics

Characteristics	Patients (n = 70)
*Age (year)*	
≤ 60	35 (50.0)
> 60	35 (50.0)
*Sex*	
Male	62 (88.6)
Female	8 (11.4)
*Smoking history*	
Never	29 (41.4)
Former or current	41 (58.6)
*Drinking history*	
Never	27 (38.6)
Former or current	43 (61.4)
*Stage (AJCC, 8th edition)*	
IIa	0 (0.0)
IIb	13 (18.6)
IIIa	7 (10.0)
IIIb	42 (60.0)
IVa	8 (11.4)
*T phase*	
T2	14 (20.0)
T3	52 (74.3)
T4a	4 (5.7)
*N phase*	
N0	14 (20.0)
N1	32 (45.7)
N2	16 (22.9)
N3	8 (11.4)
*Tumor location*	
Upper	3 (4.3)
Middle	48 (68.6)
Lower	19 (27.1)
Multiple	0 (0.0)
*Lymphadenectomy*	
Two-field	49 (70.0)
Three-field	17 (24.3)
Unknown	4 (5.7)

### Treatment

Radiation parameters were shown in Additional file 2: Table S2. All parameters, including V5 and V20 of the lungs, maximum dose of spinal cord, mean dose of heart, were higher than those in ESO-Shanghai 8 because of higher prescription dose in this study. Nearly all patients finished postoperative radiotherapy and the interruption during radiotherapy occurred in over one-third patients, most of whom were due to grade 3/4 neutropenia (19 in 70 patients, 27.1%), which was much more than that in ESO-Shanghai 8 trial (1 in 70 patients, 1.4%).

As for chemotherapy, 70% patients (49 in 70 patients) received no less than 4 cycles of concurrent chemotherapy and dose deduction occurred in four patients. (Additional file 2: Table S3) Most frequent reasons for chemotherapy suspension were 3/4 neutropenia (26 in 70 patients, 37.1%).

### Survival

**Fig. 1:**
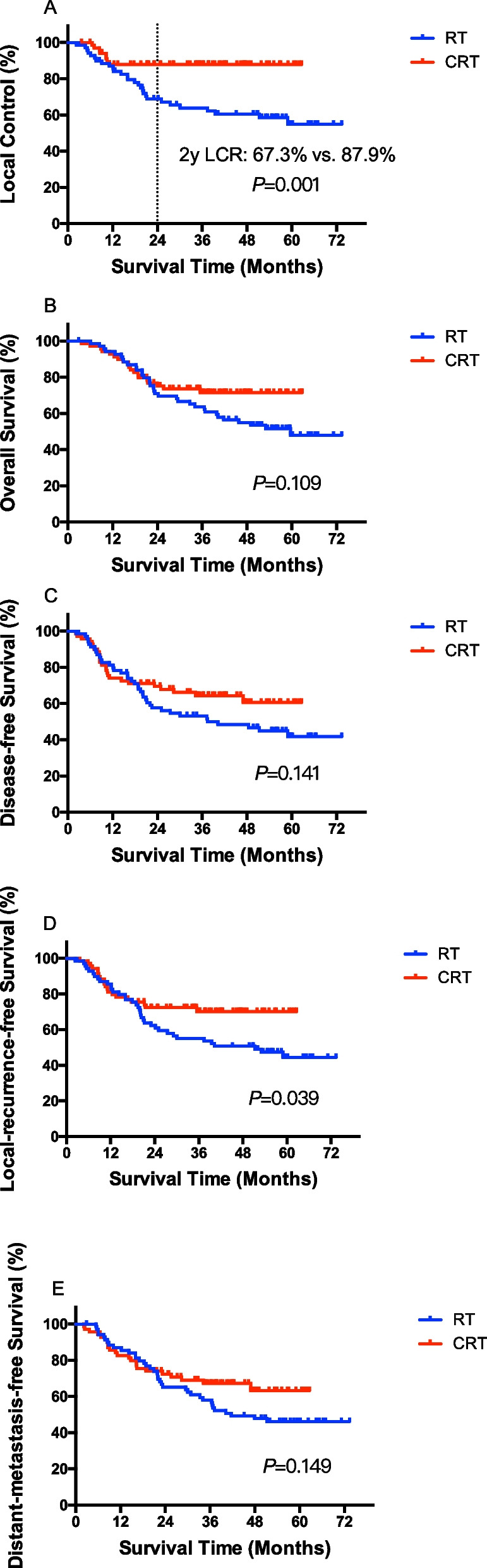
Local control rate (**A**), overall survival (**B**), disease free survival (**C**), local-regional recurrence free survival (**D**), distant metastasis free survival (**E**) of the enrolled patients in ESO-Shanghai 8 study (RT group) and this study (CRT group)

After median follow-up time of 57.5 (95% CI: 52.8–62.2) months in ESO-Shanghai 8 study and 41.1 (95% CI: 36.9–45.2) months in this study, the primary endpoint, the 2-year local control rate was 87.9% (95% CI: 83.3–92.4) in this study, which achieved the hypothesized 2-year local control rate of at least 83%. The 2-year overall survival rate were 69.6% in ESO-Shanghai 8 study and 73.6% in this study, respectively (*P* = 0.109), and 2-year disease free survival rate were 57.6% in ESO-Shanghai 8 study and 69.5% in this study, respectively (*P* = 0.141). Two-year local recurrence free survival in this study was much longer than that in ESO-Shanghai 8 study (72.4% vs. 62.3%, *P* = 0.039), and 2-year distant metastasis free survival were 65.2% in ESO-Shanghai 8 study and 72.4% in this study (*P* = 0.149). (Fig. 1).

### Failure patterns

Of the 70 patients, 21 failed in the follow-up period. We analyzed the patterns of failure as follows (Table 2). With respect to initial tumor recurrence, loco-regional recurrence was found in 8 patients (11.4%), much fewer than that in ESO-Shanghai 8 study (28 in 70 patients, 40%), most of which in mediastinal lymph node area (5 patients) and abdominal lymph node areas (3 patients), followed by anastomosis (2 patients), supraclavicular lymph node region (2 patients). Thirteen patients (18.6%) were found distant metastasis. Liver (8 patients, 11.4%) was the most frequent among metastasis sites followed by lung (3 patients, 4.3%) and bone (3 patients, 4.3%).

**Table 2:** Patterns of first treatment failure

First failure	No. of patients (%)	
	CRT group (n = 70)	RT group (n = 70)
No failure	49 (70.0)	32 (45.7)
Loco-regional failure (in field)	8 (11.4)	28 (40.0)
Anastomosis	2 (2.9)	5 (7.1)
Supraclavicular LN	2 (2.9)	10 (14.3)
Superior mediastinal and subcarinal LN	3 (4.3)	14 (20.0)
Lower mediastinal LN	2 (2.9)	3 (4.3)
Abdominal LN	3 (4.3)	3 (4.3)
Distant metastasis	13 (18.6)	18 (25.7)
Lung	3 (4.3)	10 (14.3)
Liver	8 (11.4)	5 (7.1)
Bone	3 (4.3)	6 (8.6)
LN outside radiation field	0 (0.0)	1 (1.4)
Pleura	0 (0.0)	1 (1.4)
Second primary tumor	2 (2.9)	2 (2.9)

*There will be overlapping of patients in various recurrence situations, but the denominator is 70 when we calculating the ratio

### Toxicity

Toxicities were evaluated according to CTCAE 4.0. Hematological toxicities were much more severe in chemoradiation group from our study than that in radiation-only group in ESO-Shanghai 8 study. Similarly, fatigue and gastrointestinal adverse events were more frequent in chemoradiation group than radiation-only group. As for radiation-induced toxicities, grade 2 or more pneumonitis, esophagitis and dermatitis were also more frequent in chemoradiation group than radiation-only group (27.1% vs. 20.0% for pneumonitis, 17.1% vs. 7.1% for esophagitis, 7.1% vs. 1.4% for dermatitis, respectively). Two patients died of radiation pneumonitis in chemoradiation group (Table 3).

**Table 3:** Acute treatment toxicities

Toxicity	No. of patients (%)									
	CRT group (n = 70)					RT group (n = 70)				
	Grade 1	Grade 2	Grade 3	Grade 4	Grade 5	Grade 1	Grade 2	Grade 3	Grade 4	Grade 5
*Hematological toxicity*										
Leukopenia	2 (2.9)	11 (15.7)	46 (65.7)	9 (12.9)	–	31 (44.3)	24 (34.3)	4 (5.7)	0 (0.0)	–
Neutropenia	7 (10.0)	28 (40.0)	18 (25.7)	9 (12.9)	–	12 (17.1)	5 (7.1)	1 (1.4)	0 (0.0)	–
Anemia	52 (74.3)	16 (22.9)	1 (1.4)	0 (0.0)	0 (0.0)	14 (20.0)	0 (0.0)	0 (0.0)	0 (0.0)	0 (0.0)
Thrombocytopenia	40 (57.1)	18 (25.7)	4 (5.7)	0 (0.0)	–	30 (42.9)	5 (7.1)	0 (0.0)	0 (0.0)	–
*Constitutional symptom*										
Fatigue	43 (61.4)	11 (15.7)	2 (2.9)	–	–	14 (20.0)	6 (8.6)	2 (2.9)	–	–
*GI*										
Nausea	26 (37.1)	21 (30.0)	5 (7.1)	–	–	32 (45.7)	9 (12.9)	5 (7.1)	–	–
Vomiting	16 (22.9)	6 (8.6)	1 (1.4)	0 (0.0)	0 (0.0)	6 (8.6)	5 (7.1)	1 (1.4)	0 (0.0)	0 (0.0)
*Radiation induced*										
Dermatitis	23 (32.9)	5 (7.1)	0 (0)	0 (0)	0 (0)	5 (7.1)	1 (1.4)	0 (0.0)	0 (0.0)	0 (0.0)
Esophagitis	49 (70.0)	11 (15.7)	1 (1.4)	0 (0.0)	0 (0.0)	39 (55.7)	5 (7.1)	0 (0.0)	0 (0.0)	0 (0.0)
Pneumonitis	36 (51.4)	17 (24.3)	2 (2.9)	0 (0.0)	2 (2.9)	27 (38.6)	13 (18.6)	1 (1.4)	0 (0.0)	0 (0.0)

Acute AE were defined as occurred during or within 6 months after radiotherapy

## Discussion

In this phase II clinical trial, we explored the efficacy and safety of postoperative extensive target volume irradiation with elevated radiation dose and concurrent chemotherapy. Compared with the ESO-Shanghai 8 study, the survival results of this study showed a significant improvement in the 2-year local control rate and met the hypothesized 2-year local control rate of at least 83%. Treatment related toxicity was increased due to higher treatment intensity.

Data were conflicting about the role of postoperative radiotherapy. In the clinical trials from Ténière et al. and Fok et al., no overall survival improvement was shown in the results [13, 14]. However, these studies with negative results were reported in early 1990s and the results might be biased by the two-dimensional technology and low-quality equipment of that era. Besides, in Xiao et al. study, 495 patients were randomized into postoperative radiation group and observation group. No survival benefit was shown for the entire cohort (*p* = 0.4474). However, for stage III patients, great improvement was seen in 5-year overall survival from 13.1 to 35.1% (*p* = 0.0027) but not for stage II patients (*p* = 0.6344) [4]. Similar results were provided by some large population-based studies. Schreiber et al. demonstrated there was significant improvement in 3-year overall survival from 18.2 to 28.9% (*p* < 0.001) only in stage III esophageal carcinoma (T3N1M0 or T4N0-1M0) rather than in stage IIA and IIB disease based on SEER database [6]. In Wong et al. [8] study, data from NCDB showed the addition of postoperative chemoradiation after esophagectomy would benefit the patients diagnosed with stage pT3-4Nx-0M0 or pT1-4N1-3M0 esophageal carcinoma. These data suggested that adjuvant radiotherapy still played a significant role in selected patients after radical resection of esophageal cancer, especially for advanced patients.

In our previous study (ESO-Shanghai 8), an extensive target volume to cover all high-risk recurrence areas and a total dose of 40 Gy were used in postoperative radiotherapy. The extensive target volume irradiation was feasible in dose distribution and the toxicity was tolerable, but the in-field recurrence rate remained high [10]. In the studies from Xiao et al. and Chen et al., the total dose for postoperative radiotherapy was around 50 Gy [4, 5, 9, 15]. We believe that lower dose may be an explanation for higher recurrence rate in our previous study. Therefore we elevated the radiation dose to 45 Gy considering the feasibility of planning and safety, and the local control rate was significantly improved in this study.

In the ESO-Shanghai 8 study, we found that the distant metastasis rate was relatively high [10]. The addition of concurrent chemotherapy into postoperative radiotherapy was also an approach to improve the survival. In Chen et al. [12] study, compared with the radiotherapy alone, concurrent chemoradiation had significantly lower rate of distant metastasis. Similar results could be found in the study of Hwang et al. [16]. Therefore, we added weekly concurrent TC chemotherapy to make the radiotherapy more sensitive and to kill micro-metastases that cannot be detected by imaging.

In this study, the toxicity was more frequent and severe than that in ESO-Shanghai 8 study, which was partly due to the addition of chemotherapy [10]. Similar to the data in Chen's article, hematological toxicity was significantly increased in chemoradiation group than the radiation group [12]. Besides, the severe toxicities were also caused by the large target volume and the increased dose of radiotherapy. Doses of organs as risk, such as lung and heart were higher and radiotherapy-related toxicities were also correspondingly increased. In Qiao's retrospective study, regional target volume did not compromise the survival than extensive target volume in postoperative radiotherapy [17]. Besides, in the recent phase III study, Prof. Xiao’s team [18] adapted regional target volume and no grade 4 or 5 toxicities were reported. Regional target volume could optimize the dose distribution of normal tissues, and also provide the possibility for higher dose radiotherapy of around 50 Gy, thereby reducing the risk of local recurrence in high-risk areas.

Compared with the surgery data of our center, the rate of recurrence of supraclavicular and mediastinal lymph nodes was reduced in the chemoradiotherapy group in this study (Supraclavicular: 6.5% vs. 2.9%; Mediastinal: 12.3% vs. 5.7%) while no difference was observed in the rate of recurrence of abdominal lymph nodes. (Abdominal: 5.0% vs. 4.3%) [19].

Therefore, based on the results of this study, we designed another phase II clinical study (ESO-Shanghai 17, NCT 04764227), using targeted high-risk regional areas (supraclavicular, upper and middle mediastinal lymph node areas) as clinical target volume for radiotherapy and also concurrent with weekly TC chemotherapy in patients after radical resection of T3-4NxM0 or TxN + M0 esophageal cancer, which was expected to reduce the occurrence of treatment-related toxicity without reducing the local control rate and lowering the disease-free survival.

As a single-arm study, there were limitations in study design. We could only compare the results of this study with historical studies, which may be potentially biased by different accrual time/sites and patient characteristics across studies. Besides, many studies applied immune checkpoint inhibitors to neoadjuvant therapy followed by surgery in recent years, and the pathological complete response rates reported in various studies were inspiring. For these patients, whether postoperative chemoradiotherapy is still meaningful and what is the most suitable target volume for radiation needed further research.

In all, postoperative extensive target volume irradiation with elevated radiation dose and concurrent chemotherapy was effective. Treatment related toxicity was increased due to higher treatment intensity. We hope postoperative chemoradiotherapy with high-risk regional target volumes in future clinical trials could decrease the treatment-related toxicity rate without reducing the efficacy.

## Supplementary Information

**Additional file 1**: **Fig. S1**. Planning target volume of extensive target volume irradiation in this study. Clinical target volume (CTV) included tumor bed, anastomosis site, bilateral supraclavicular region, all mediastinal lymph node site, left gastric and celiac trunk lymph node site. The superior, inferior, anterior, posterior and lateral borders of planning target volume were 1 cm beyond CTV.

**Additional file 2**. **Table S1.** Normal Organ Dose Restrictions. **Table S2.** Radiotherapy Parameters. **Table S3.** Chemotherapy Parameters.

## Data Availability

The datasets used and analyzed during the current study are available from the corresponding author on reasonable request.
