# Supplementary figures and images for: Extensive clinical target volume in postoperative chemoradiotherapy for esophageal squamous cell carcinoma: a phase II clinical trial (ESO-Shanghai 9)
Source: Radiat Oncol. 2023 Feb 7;18:26. doi: 10.1186/s13014-023-02211-w (PMC9903423; doi:10.1186/s13014-023-02211-w)

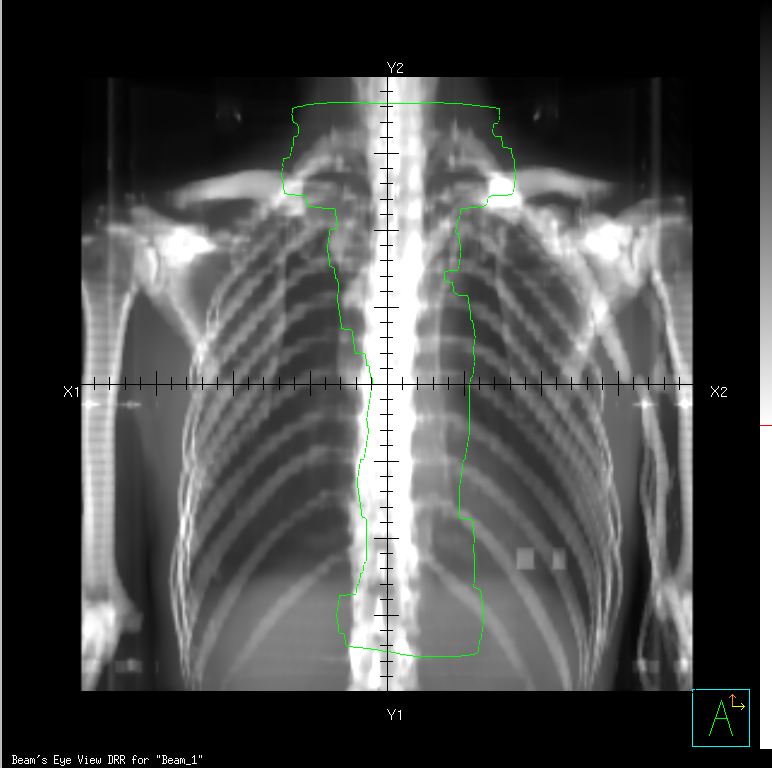

Supplement: Supplementary file 1 — Additional file 1: Fig. S1. Planning target volume of extensive target volume irradiation in this study. Clinical target volume (CTV) included tumor bed, anastomosis site, bilateral supraclavicular region, all mediastinal lymph node site, left gastric and celiac trunk lymph node site. The superior, inferior, anterior, posterior and lateral borders of planning target volume were 1 cm beyond CTV. [file 13014_2023_2211_MOESM1_ESM.tiff]
