# Supplementary material for: Extensive clinical target volume in postoperative chemoradiotherapy for esophageal squamous cell carcinoma: a phase II clinical trial (ESO-Shanghai 9)
Source: Radiat Oncol. 2023 Feb 7;18:26. doi: 10.1186/s13014-023-02211-w (PMC9903423; doi:10.1186/s13014-023-02211-w)
Supplement: Supplementary file 2 — Additional file 2. Table S1. Normal Organ Dose Restrictions. Table S2. Radiotherapy Parameters. Table S3. Chemotherapy Parameters. [file 13014_2023_2211_MOESM2_ESM.pdf]

Risk organs	Contour regulation	Dose restriction
Spinal cord	All the layers of CT scan have to be contoured and the margin of vertebra tube can be regarded as that of planning organ at risk volume.	Highest point dose less than 45Gy
Lung	It is allowed to use automatic tools in the delineation of margin of lungs. (Trachea and bronchia must be contoured manually)	V20 of lung (PTV excluded) has to be less than 30%, V5 less than 65%.
Heart	The superior margin of heart consists of right atrium and right ventricle, pulmonary artery trunk, ascending main aorta and superior vena cava excluded. The inferior margin is at the level of heart apex.	V30 of heart has to be less than 45% and the mean dose has to be less than 35Gy.

Table S1. Normal Organ Dose Restrictions

Table S2. Radiotherapy Parameters

Radiotherapy parameters	No. of patients (%)	
	CRT group (n=70)	RT group (n=70)
Finished radiotherapy	64 (91.4)	68 (97.1)
Interruption during radiotherapy	27 (38.6)	4 (5.7)
PTV, cm^3	1327.7 (1281.7, 1373.7)	1273.2 (1225.0, 1321.4)
Lung V5, %	65.9 (65.0, 66.7)	64.6 (62.8, 66.4)
Lung V20, %	27.5 (26.9, 28.1)	26.2 (24.1, 28.3)
Spinal cord maximum dose, Gy	44.2 (43.9, 44.5)	40.9 (40.5, 41.3)
Mean heart dose, Gy	32.5 (31.7, 33.3)	30.3 (29.6, 31.0)

Table S3. Chemotherapy Parameters

Concurrent cycles	No. of patients (%)
1	6 (8.6)
2	4 (5.7)
3	11 (15.7)
4	19 (27.1)
5	30 (42.9)
Deduction in dose	4 (5.7)
